# Reliability, Validity, and Classification Accuracy of the DSM-5 Diagnostic Criteria for Gambling Disorder and Comparison to DSM-IV

**DOI:** 10.1007/s10899-015-9573-7

**Published:** 2015-09-25

**Authors:** Randy Stinchfield, John McCready, Nigel E. Turner, Susana Jimenez-Murcia, Nancy M. Petry, Jon Grant, John Welte, Heather Chapman, Ken C. Winters

**Affiliations:** 1 Department of Psychiatry, University of Minnesota Medical School, Minneapolis, MN USA; 2 Healthy Horizons Consulting, Toronto, ON Canada; 3 Centre for Addiction and Mental Health, University of Toronto, Toronto, ON Canada; 4 Department of Psychiatry, University Hospital of Bellvitge-IDIBELL, Barcelona, Spain; 5 CIBEROBN, Barcelona, Spain; 6 University of Connecticut Health Center, Farmington, CT USA; 7 Department of Psychiatry and Behavioral Neuroscience, University of Chicago, Chicago, IL USA; 8 Research Institute on Addictions, Buffalo, NY USA; 9 Louis Stokes Cleveland VA Medical Center, Cleveland, OH USA

**Keywords:** Gambling disorder, Diagnosis, DSM-5, DSM-IV, Classification accuracy

## Abstract

The DSM-5 was published in 2013 and it included two substantive revisions for gambling disorder (GD). These changes are the reduction in the threshold from five to four criteria and elimination of the illegal activities criterion. The purpose of this study was to twofold. First, to assess the reliability, validity and classification accuracy of the DSM-5 diagnostic criteria for GD. Second, to compare the DSM-5–DSM-IV on reliability, validity, and classification accuracy, including an examination of the effect of the elimination of the illegal acts criterion on diagnostic accuracy. To compare DSM-5 and DSM-IV, eight datasets from three different countries (Canada, USA, and Spain; total N = 3247) were used. All datasets were based on similar research methods. Participants were recruited from outpatient gambling treatment services to represent the group with a GD and from the community to represent the group without a GD. All participants were administered a standardized measure of diagnostic criteria. The DSM-5 yielded satisfactory reliability, validity and classification accuracy. In comparing the DSM-5 to the DSM-IV, most comparisons of reliability, validity and classification accuracy showed more similarities than differences. There was evidence of modest improvements in classification accuracy for DSM-5 over DSM-IV, particularly in reduction of false negative errors. This reduction in false negative errors was largely a function of lowering the cut score from five to four and this revision is an improvement over DSM-IV. From a statistical standpoint, eliminating the illegal acts criterion did not make a significant impact on diagnostic accuracy. From a clinical standpoint, illegal acts can still be addressed in the context of the DSM-5 criterion of lying to others.

## Introduction

Accurate diagnosis of gambling disorder (GD) is important in order to measure the prevalence of GD in the general population, manage public health efforts, diagnose patients in clinical settings, and measure treatment outcome. A concise list of cardinal symptoms of GD is also important as a tool to make the general public aware of the warning signs of GD.

Diagnostic criteria for GD were first introduced in 1980, under the diagnosis of pathological gambling (PG) in DSM-III (American Psychiatric Association 1980) and were revised in 1987 for DSM-III-R (American Psychiatric Association 1987; Lesieur 1988), and again in 1994 for DSM-IV (American Psychiatric Association 1994), and most recently in 2013 for DSM-5 (American Psychiatric Association 2013). The original criteria and subsequent revisions were written by committees of experts based upon a number of factors including a review of the literature and their clinical experience and expertise. Lesieur and Rosenthal’s (1991) literature review for the DSM-IV committee found little data regarding the diagnostic criteria other than clinician opinions and anecdotal reports. While the DSM-IV is not an exhaustive list of PG symptoms, it is thought to include symptoms that are sufficient to provide accurate diagnosis (Gebauer et al. 2010). Subsequent to the publication of DSM-IV, there were only a small number of empirical studies on the reliability, validity, and classification accuracy of diagnostic criteria for PG (National Research Council 1999; Petry et al. 2013; Zimmerman et al. 2006).

Research to date on the classification accuracy of DSM-IV diagnostic criteria for PG has shown that most diagnostic errors are false negatives (i.e., concluding that the person *doesnot* have the disorder, when in fact they *do*), and these classification errors occur just below the standard threshold of five criteria (Jimenez-Murcia et al. 2009; Lesieur and Rosenthal 1991; Stinchfield 2003; Stinchfield et al. 2005; Zimmerman et al. 2006). As a result of these diagnostic errors, some investigators have suggested lowering the cut score from five to four (Jimenez-Murcia et al. 2009; Stinchfield 2003; Stinchfield et al. 2005) while others have suggested eliminating one or more criteria (Strong and Kahler 2007; Zimmerman et al. 2006).

The DSM-5 includes the following revisions: (a) renaming the disorder from PG to GD; (b) reclassifying from impulse control disorders to substance-related and addictive disorders; (c) elimination of the criterion “has committed illegal acts such as forgery, fraud, theft, or embezzlement to finance gambling”; (d) reducing the threshold for diagnosis from five criteria to four criteria; and (e) specifying that symptoms occur within a 12 month time period. The decision to eliminate one of the ten criteria and reduce the threshold from five to four criteria was based on empirical data. The reduction in threshold from five to four criteria was based on three studies in three different countries, USA (Stinchfield 2003), Canada (Stinchfield et al. 2005), and Spain (Jimenez-Murcia et al. 2009). All three studies found a modest improvement in classification accuracy using a cut score of four, and most importantly, a reduction in false negatives. Six of the nine DSM-5 diagnostic criteria are unchanged from DSM-IV and three criteria have minor revisions to the wording, such as inserting the word “often” in the preoccupation criterion, but the content and meaning of these criteria remain unchanged.

The empirical rationale for the elimination of the illegal acts criterion was based primarily on two studies which found low prevalence rates for this criterion (Strong and Kahler 2007; Zimmerman et al. 2006). These two studies note that the illegal acts criterion is rarely endorsed in the absence of other criteria and thus does not add to diagnostic accuracy. Research has demonstrated that illegal activity secondary to gambling is associated with more severe gambling symptomatology (Ledgerwood et al. 2007). A similar conclusion was reached in a more recent study in Spain (Granero et al. 2014). Illegal acts (i.e., arrests) were included as diagnostic criteria when PG was first introduced in 1980 in DSM-III and has been present in DSM-III-R and DSM-IV. However, the illegal acts criterion is not without its problems. The illegal acts criterion can be misunderstood by interviewees and therefore may also be the most underreported criterion. For example, many problem gamblers write bad checks, but they may not consider that act to be “illegal”, particularly if they plan to put money in the checking account later to cover the bad check and if they did not suffer any adverse consequences from it, such as being arrested. This criterion, more than any other criteria, requires clarification and probing questions during the diagnostic interview to establish whether an act, such as writing a bad check, satisfies the criterion of illegal acts. Some individuals may not report illegal behavior in a clinical assessment for fear of the implications of admitting to an illegal act. Anecdotally, clinicians report that some individuals who do not report illegal activity at the initial diagnostic assessment will later in the course of treatment disclose illegal activity that they engaged in to fund their gambling or pay gambling debts. This underreporting may also contribute to low prevalence rates for this criterion, which is partly why it was eliminated as a stand-alone criterion in DSM-5.

Temcheff et al. (2011) compared DSM-IV and DSM-5 in a large sample (n = 19,942) of US college athletes and found a statistically significant increase in the proportion of male participants diagnosed with GD using DSM-5 (3.4 % DSM-IV vs. 4.3 % DSM-5). The authors did not compare classification accuracy between DSM-IV and DSM-5. In France, Denis et al. (2012) compared DSM-IV to DSM-5 in a small sample of clients (n = 161) presenting for addictions treatment. They found that DSM-IV and DSM-5 yielded similar prevalence rates that were not statistically significantly different and exhibited a high degree of agreement (*kappa* = .94). They also found that some criteria were better at discriminating between pathological and non-pathological gamblers than others, namely “repeated unsuccessful efforts to stop”, “chasing losses”, “lying”, and “jeopardized or lost a significant relationship or job”. Like the Temcheff et al. (2011) study above, this study also did not provide classification accuracy estimates.

In another evaluation of DSM-5 criteria for GD, Petry et al. (2013) examined the proposed criteria in five US samples drawn from a general population, gambling patrons, and treatment studies, totaling 3710 participants. Internal consistency of the nine criteria yielded a Cronbach’s *alpha* = .95. The illegal acts criterion loaded adequately in the principal components analysis, however, it had the lowest factor loading of the ten criteria, rarely was present in the absence of other criteria, and its absence did not significantly diminish diagnostic accuracy. Classification accuracy of the DSM-5 criteria, using the DSM-IV criteria as the reference standard, resulted in *sensitivity* = 100 %, *specificity* = 98 %, and *hit rate* = 98 %. The cut score of four performed as well or better than a cut score of five in all samples. The authors acknowledge that a limitation of this study was that the reference standard (a measure of DSM-IV) was not independent of the test (a measure of DSM-5); in fact, it was from the same instrument (NODS), and therefore accuracy results may have been inflated due to this non-independence. The reference standard against which the test is evaluated must be independent of the test itself (Gambino 2012). The methodological limitations of these studies along with the fact that diagnostic test results can vary as a function of small sample characteristics suggests that additional testing of DSM-5 is warranted. This current study improves on the studies reviewed above in two ways: (1) the current study uses a reference standard (group membership) that is largely independent from the test (DSM-5); and (2) this study draws on larger and more diverse samples from the USA, Canada and Spain.

The following three questions will be addressed in the present study: (1) What is the reliability, validity, and classification accuracy of the DSM-5 diagnostic criteria for GD? (2) Is the DSM-5 revision an improvement over DSM-IV in terms of reliability, validity and classification accuracy? and (3) How many individuals will not be diagnosed with GD with the illegal acts criterion removed from DSM-5?

## Methods

### Participants

This study analyzed eight existing datasets. These eight datasets were selected based upon the following two inclusion criteria: (a) DSM-IV diagnostic criteria were assessed; and (b) the reference standard for classification accuracy was group membership, that is, participants were recruited from clinical and community settings. Participants in each dataset are described in Table 1 and further details can be found in the original source cited. The datasets included adults recruited from clinical and community populations in Canada, USA, and Spain. Inclusion criteria for participants in the clinical samples were that they were admitted to a gambling treatment; and were 18 years of age or older. Inclusion criteria for participants in the community samples were that they were 18 years of age or older and had gambled at least once in past year. For the Canadian and USA datasets, the participants had to understand the English language, and for the Spanish samples, the participants had to understand the Spanish language. In total, across all eight datasets, the combined sample was 3247; and there were 1871 males and 1350 females and 26 with unknown gender. There were 1431 clinical participants and 1816 community participants. Each dataset was obtained from the principal investigator by permission and each study had received prior human subjects’ approval.

**Table 1:** Source, demographics and group membership sample size for each dataset

Dataset	Gender Male n (%) Female n (%)	Age Range Mean	Sample size by group Clinical n (%) Community n (%)
(1) Stinchfield et al. (2012); Ontario; n = 422	208 (49)	18–75	200 (47)
	214 (51)	44.3	222 (53)
(2) Stinchfield et al. (2012); Minnesota; n = 212	91 (43)	18–68	92 (43)
	121 (57)	42.2	120 (57)
(3) Jimenez-Murcia et al. (2012); Spain; n = 282	252 (90)	17–79	232 (82)
	28 (10)	43.6	50 (18)
(4) Jimenez-Murcia et al. (2009); Spain; n = 569	522 (92)	17–88	286 (50)
	47 (8)	40	283 (50)
(5) Stinchfield et al. (2007); Minnesota; n = 135	51 (38 %)	20–63	91 (67)
	84 (62 %)	42	44 (33)
(6) Stinchfield et al. (2007); Minnesota; n = 175	71 (41)	18–67	150 (86)
	104 (59)	42	25 (14)
(7) Stinchfield et al. (2005); Ontario; n = 390	170 (44)	19–78	121 (31)
	196 (50)	44	269 (69)
(8) Stinchfield (2003); Minnesota; n = 1062	506 (48)	18–90	259 (24)
	556 (52)	44	803 (76)

### Instrument

As the DSM-5 revision includes elimination of one criterion and lowering the cut score from five to four, the DSM-5 can be evaluated with existing DSM-IV datasets. All eight datasets identified above used Stinchfield’s measure of DSM-IV diagnostic criteria for PG which is part of the Gambling Behavior Interview (GBI; Stinchfield 2001, 2003; Stinchfield et al. 2005) and the Gambling Treatment Outcome Monitoring System (GAMTOMS; Stinchfield et al. 2007). The GBI is a 106-item structured interview that measures gambling frequency, time and money spent gambling, South Oaks Gambling Screen (SOGS; Lesieur and Blume 1987, 1993), DSM-IV diagnostic criteria, research diagnostic criteria, and demographics (Stinchfield et al. 2005; Stinchfield 2014). The GBI was developed as a research diagnostic interview and therefore it includes other measures of PG, such as the SOGS, Problem Gambling Severity Index (PGSI) from the Canadian Problem Gambling Inventory (CPGI; Ferris and Wynne 2001) and Gamblers Anonymous 20 questions (GA-20). The GAMTOMS is a multi-instrument and multidimensional gambling treatment outcome assessment battery (Stinchfield et al. 2007; Stinchfield 2014).

DSM-IV diagnostic criteria for PG are measured with ten items, one for each criterion, paraphrased from the ten DSM-IV diagnostic criteria for PG. See “Appendix” for a copy of Stinchfield’s measure of DSM-IV diagnostic criteria of PG. This measure has demonstrated satisfactory reliability with internal consistency estimates of Cronbach’s *alpha* = .92 for a combined community and gambling treatment sample (Stinchfield et al. 2005), and temporal stability as measured by 1-week test–retest was *intraclass correlation* (*ICC*) = .78 (Stinchfield et al. 2007). In terms of convergent validity, the DSM-IV PG scale was correlated with the SOGS *r* = .90 (Stinchfield et al. 2005). In terms of classification accuracy, using the standard DSM-IV cut score of five to indicate a diagnosis of PG (APA 1994), using a reference standard of group membership (clinical vs. community), yielded a hit rate = .91, sensitivity = .83, and specificity = .98, all of which are satisfactory (Stinchfield et al. 2005). The two datasets from Spain used a Spanish translation of the Stinchfield DSM-IV measure and it showed similar evidence of satisfactory reliability, validity and classification accuracy (Jimenez-Murcia et al. 2009).

In order to measure convergent validity, the DSM-5 was correlated with other measures of gambling problem severity included in these eight datasets. These convergent validity measures include the SOGS, PGSI, gambling participation, and gambling-related financial problems (see Table 3 for convergent validity measures in each dataset) that are included in the GBI (Stinchfield et al. 2005). Discriminant validity was measured by correlations of DSM-5 with demographic variables that have exhibited low or no correlation with PG.

### Procedures

This study draws on eight existing datasets and procedures were similar but not identical across all eight studies and reports from each study can be consulted for descriptions of procedures. In general, for clinical samples, clients of outpatient gambling treatment services were recruited via flyers and then in-person interviews were conducted by research staff within the first few days following admission to treatment. For community samples, a number of methods were used to recruit participants, such as flyers and notices on community bulletin boards, and participants contacted research staff to set up an in-person interview. In-person interviews were conducted with the standardized measure of DSM-IV described above that was part of a longer assessment, either the GBI or GAMTOMS. In two studies the community sample was administered the DSM-IV measure via telephone interview (Stinchfield 2003; Stinchfield et al. 2005). Participants were offered a gift card as remuneration for their time.

### Data Analyses

Data analyses were conducted on each of the eight datasets. The eight datasets were not merged due to the data coming from different countries and at different times. First, reliability, validity, and classification accuracy of the DSM-5 diagnostic criteria were computed. Second, the DSM-5 psychometric properties were compared to the DSM-IV psychometric properties. Third, the number of participants not diagnosed with GD when the illegal acts criterion was removed (keeping the cut score constant at four) was computed.

Reliability was examined by measuring internal consistency (Cronbach’s alpha) and temporal stability (1-week test–retest) of DSM-IV and DSM-5 diagnostic criteria. Internal consistency is evident if Cronbach’s coefficient alpha is >.70. Two of the eight datasets included a 1-week test–retest procedure and ICC coefficients were computed to measure temporal stability. Temporal stability is evident if the test–retest ICC coefficient is >.70 (Cichetti 1994).

Validity was examined using measures of convergent and discriminant validity. Convergent validity was examined by measuring the correlations between the total DSM-5 score and other measures of gambling problem severity, including SOGS, GA-20, PGSI, time spent gambling, money spent gambling, and gambling frequency. Convergent validity is evident if correlation coefficients are *r* > .30 (Cichetti 1994). Discriminant validity was examined by measuring the relationship between the DSM-5 total score and variables that have been shown to have low or no correlation with PG, including gender, age, marital status, educational level, income, and employment status. Discriminant validity is evident if correlation coefficients are *r* < .10 (Cichetti 1994).

Classification accuracy was measured by computing diagnostic statistics of sensitivity, specificity, false negative rate, false positive rate, positive predictive power, negative predictive power, hit rate, and base rate (Baldessarini et al. 1983; Fleiss 1981; Friedman and Cacciola 1998). In order to demonstrate satisfactory classification accuracy the hit rate, sensitivity, and specificity must all be .80 or greater (DiStefano and Morgan 2011; Glascoe 2005). In order to demonstrate superiority over DSM-IV, classification accuracy indices for the DSM-5 must be higher than those for DSM-IV.

To compute classification accuracy a reference or “gold” standard is used against which to compare the test. There is no consensus among investigators about what to use for a reference standard for diagnosing GD, so investigators have used alternate measures, such as the SOGS or a standardized diagnostic interview, however, neither of these are independent of DSM-IV diagnostic criteria. In the current study, to create a reference standard to serve as a proxy for a “gold” reference standard, group membership in either a clinical or community sample served as the reference standard (Faraone and Tsuang 1994; Gambino 2006, 2012). The clinical group was comprised of patients in gambling treatment and the community group was comprised of adults from the general population. It was assumed that patients in gambling treatment have the disorder and adults from the general population are free of the disorder. While this is a fairly safe assumption, there may be a small number of patients in treatment who do not satisfy the DSM criteria for GD, and likewise, there may be a small number of members of the general population who meet DSM criteria for GD. Nevertheless, this method has proven to be useful in previous research on the classification accuracy of DSM-IV (Jimenez-Murcia et al. 2009; Stinchfield 2003; Stinchfield et al. 2005, 2007). The use of group membership as the reference standard is the main difference between this study and prior evaluations of DSM-5 (Denis et al. 2012; Petry et al. 2013; Temcheff et al. 2011) and this group membership as reference standard is considered an improvement in methodology because it has greater independence from the test (DSM-5 measure) than the reference standard used in the prior evaluations (DSM-IV measure).

## Results

### Reliability

Reliability results are shown in Table 2. All eight datasets yielded Cronbach alphas that were >.70, and six out of eight datasets yielded Cronbach alphas >.90, indicating excellent internal consistency. For comparison purposes, the same analyses were computed for the DSM-IV, and the results indicate that the DSM-5 is slightly more internally consistent than DSM-IV. Two datasets (#5 and #6 in Table 1) included a 1-week test–retest and the ICC for DSM-IV was ICC = .74 and .76 and for DSM-5 the ICC was .71 for both datasets. Both DSM-IV and DSM-5 exhibited ICCs above the criterion of .70.

**Table 2:** Reliability of DSM-IV and DSM-5

Dataset	Internal consistency Cronbach’s coefficient alpha		Temporal stability One week Test–retest Intraclass correlation (ICC)	
	DSM-IV	DSM-IV	DSM-IV	DSM-5
(1) Stinchfield et al. (2012); Ontario; n = 422	.95	.95	NA	NA
(2) Stinchfield et al. (2012); Minnesota; n = 212	.94	.94	NA	NA
(3) Jimenez-Murcia et al (2012); Spain; n = 282	.87	.87	NA	NA
(4) Jimenez-Murcia et al. (2009); Spain; n = 569	.94	.94	NA	NA
(5) Stinchfield et al. (2007); Minnesota; n = 135	.94	.94	.74	.71
(6) Stinchfield et al. (2007); Minnesota; n = 175	.88	.88	.76	.71
(7) Stinchfield et al. (2005); Ontario; n = 390	.94	.94	NA	NA
(8) Stinchfield (2003); Minnesota; n = 1062	.98	.98	NA	NA

*NA* not available

### Validity

Convergent and discriminant validity coefficients for DSM-IV and DSM-5 are shown in Table 3. Results show moderate to high correlations with concurrent gambling problem severity measures, ranging from *r* = .21 to *r* = .97. The majority of these correlations are above the criterion of r > .30 and are identical between DSM-5 and DSM-IV. Evidence of discriminant validity of DSM-5 was exhibited by low correlations with variables purported to be unrelated to problem gambling, such as age and gender, ranging from *r* = .00 to *r* = .40. The majority of these correlations are at or below the criterion of r < .10 and are identical between DSM-5 and DSM-IV.

**Table 3:** Convergent and discriminant validity of DSM-IV and DSM-5

Dataset	Convergent and discriminant validity variable	DSM-IV	DSM-5
(1) Stinchfield et al. (2012); Ontario; n = 422	Convergent validity variables		
	SOGS	.93	.93
	PGSI	.95	.95
	GA-20	.95	.95
	Gambling frequency	.54	.54
	Money spent gambling	.25	.25
	Time spent gambling	.44	.43
	Days gambling in past month	.40	.39
	Money spent gambling in a typical month	.24	.24
	Discriminant validity variables		
	Sex	.17	.17
	Age	.15	.15
	Race	.12	.11
	Marital status	.23	.22
	Educational level	−.27	−.26
	Employment status	−.25	−.23
	Personal income	−.06	−.05
(2) Stinchfield et al. (2012); Minnesota; n = 212	Convergent validity variables		
	SOGS	.95	.94
	PGSI	.96	.96
	GA-20	.96	.96
	Gambling frequency	.46	.46
	Money spent gambling	.42	.40
	Time spent gambling	.21	.21
	Days gambling in past month	.60	.59
	Money spent gambling in a typical month	.44	.42
	Discriminant validity variables		
	Sex	−.01	−.01
	Age	.40	.40
	Race	.18	.18
	Marital status	.18	.18
	Educational level	−.17	−.17
	Employment status	−.13	−.14
	Personal income	.09	.08
	Household income	.00	−.02
(3) Jimenez-Murcia et al. (2012); Spain; n = 282	Convergent validity variables		
	SOGS	.86	.86
	PGSI	.89	.89
	GA-20	.90	.89
	Gambling frequency	.50	.49
	Days gambling in past month	.35	.34
	Discriminant validity variables		
	Sex	−.03	−.01
	Age	−.27	−.26
	Race	.11	.10
	Marital status	.11	.10
	Educational level	.00	−.02
	Employment status	.15	.14
	Personal income	−.29	−.28
(4) Jimenez-Murcia et al. (2009); Spain; n = 569	Convergent validity variables		
	Gambling frequency	.50	.50
	Largest amount gambled in 1 day	.85	.85
	SOGS	.95	.95
	Discriminant validity variables		
	Sex	.00	.00
	Age	−.01	.00
	Marital status	−.11	−.11
	Educational level	−.21	−.21
	Employment status	.14	.14
(5) Stinchfield et al. (2007); Minnesota; n = 135	Convergent validity variables		
	SOGS	.93	.92
	Gambling frequency	.57	.57
	Number of days gambling in past 30 days	.53	.51
	Largest amount gambled in 1 day	.22	.21
	Gambling debt from past 12 months	.36	.36
	Number of financial problems	.69	.67
	Discriminant validity variables		
	Gender	.28	.27
	Age	.21	.23
	Marital status	.18	.18
	Level of education	−.35	−.36
	Employment status	−.13	−.14
	Personal income	−.11	−.12
(6) Stinchfield et al. (2007); Minnesota; n = 175	Convergent validity variables		
	SOGS	.88	.87
	Gambling frequency	.50	.50
	Number of financial problems	.65	.62
	Discriminant validity variables		
	Gender	.11	.10
	Age	.04	.04
	Race	.05	.05
	Marital status	.19	.16
	Level of education	.02	.00
	Employment status	.20	.18
	Personal income	.20	.18
(7) Stinchfield et al. (2005); Ontario; n = 390	Convergent validity variables		
	SOGS	.97	.97
	Gambling frequency	.71	.71
	Largest amount gambled in 1 day	.65	.66
	Discriminant validity variables		
	Gender	−.08	−.08
	Age	−.18	−.18
	Marital status	.05	.05
	Level of education	−.05	−.06
	Employment status	−.07	−.07
	Personal income	−.34	−.34
(8) Stinchfield (2003); Minnesota; n = 1062	Convergent validity variables		
	SOGS	.97	.97
	Gambling frequency	.71	.71
	Largest amount gambled in 1 day	.65	.65
	Discriminant validity variables		
	Gender	−.08	−.08
	Age	−.18	−.18
	Marital status	.05	.05
	Level of education	−.05	−.06
	Employment status	−.07	−.07
	Personal income	−.34	−.34

### Classification Accuracy

Standard indices of classification accuracy of DSM-5 are shown in Table 4. Both DSM-5 and DSM-IV show evidence of accuracy using the reference standard of group membership (gambling treatment vs. community). All of the DSM-5 classification accuracy coefficients for hit rate, sensitivity, and specificity are above the criterion of .80 and six of the eight datasets exhibited coefficients above .90 indicating excellent classification accuracy.

**Table 4:** Classification accuracy of DSM-IV and DSM-5

Source of data	Classification indices	DSM-IV	DSM-5
(1) Stinchfield et al. (2012); Ontario; n = 422; treatment = 200; community = 222	Base rate	.47	.47
	Hit rate	.95	.94
	Sensitivity	.96	.98
	Specificity	.94	.91
	FPR	.06	.09
	FNR	.04	.02
	PPP	.93	.91
	NPP	.96	.98
(2) Stinchfield et al. (2012); Minnesota; n = 212; treatment = 92; community = 120	Base rate	.43	.43
	Hit rate	.94	.90
	Sensitivity	.99	1.00
	Specificity	.90	.83
	FPR	.10	.17
	FNR	.01	.00
	PPP	.88	.81
	NPP	.99	1.00
(3) Jimenez-Murcia et al. (2012); Spain; n = 282; treatment = 232; community = 50	Base rate	.82	.82
	Hit rate	.81	.90
	Sensitivity	.77	.88
	Specificity	1.00	1.00
	FPR	0	0
	FNR	.23	.13
	PPP	1.00	1.00
	NPP	.49	.63
(4) Jimenez-Murcia et al. (2009); Spain; n = 569; treatment = 286; community = 283	Base rate	.50	.50
	Hit rate	.95	.97
	Sensitivity	.92	.95
	Specificity	.99	.99
	FPR	.01	.01
	FNR	.08	.05
	PPP	.99	.99
	NPP	.92	.96
(5) Stinchfield et al. (2007); Minnesota; n = 135; treatment = 91; community = 44	Base rate	.33	.33
	Hit rate	.95	.95
	Sensitivity	.97	.98
	Specificity	.93	.91
	FPR	.07	.09
	FNR	.03	.02
	PPP	.97	.96
	NPP	.93	.95
(6) Stinchfield (2003); Minnesota; n = 175; treatment = 150; community = 25	Base rate	.86	.86
	Hit rate	.95	.97
	Sensitivity	.95	.97
	Specificity	.96	.96
	FPR	.04	.04
	FNR	.05	.03
	PPP	.99	.99
	NPP	.77	.86
(7) Stinchfield et al. (2005); Ontario; n = 390; treatment = 121; community = 269	Base rate	.31	.31
	Hit rate	.94	.96
	Sensitivity	.83	.92
	Specificity	.99	.98
	FPR	.01	.02
	FNR	.17	.08
	PPP	.97	.96
	NPP	.93	.96
(8) Stinchfield (2003); Minnesota; n = 1062; treatment = 259; community = 803	Base rate	.25	.25
	Hit rate	.98	.99
	Sensitivity	.95	.97
	Specificity	.996	.993
	FPR	.004	.007
	FNR	.05	.03
	PPP	.99	.98
	NPP	.98	.99

*FPR* false positive rate, *FNR* false negative rate, *PPP* positive predictive power, *NPP* negative predictive power (Baldessarini et al. 1983; Fleiss 1981; Friedman and Cacciola 1998)

A comparison of prevalence rates of DSM-5 and DSM-IV is presented in Table 5. In all eight datasets the prevalence rate for GD was slightly higher for DSM-5 as compared to DSM-IV, but none of the datasets showed a statistically significant difference at alpha = .01.

**Table 5:** Comparison of GD prevalence rates between DSM-IV and DSM-5

Source of data: Investigator, date, and type of study	DSM-IV	DSM-5	Difference in prevalence		
			Difference	z	*p*
(1) Stinchfield et al. (2012); Ontario; n = 422	205/422 = .486	215/422 = .509	.023	−.69	.49
(2) Stinchfield et al. (2012); Minnesota; n = 212	103/212 = .486	113/212 = .533	.047	−.97	.33
(3) Jimenez-Murcia et al. (2012); Spain n = 282	179/282 = .635	203/282 = .720	.085	−2.16	.03
(4) Jimenez-Murcia et al. (2009); Spain; n = 569	265/569 = .466	277/569 = .487	.021	−.71	.48
(5) Stinchfield et al. (2005); Minnesota; n = 135	87/130 = .669	89/130 = .685	.016	−.27	.79
(6) Stinchfield (2003); Minnesota; n = 175	144/175 = .823	147/175 = .840	.017	−.43	.67
(7) Stinchfield et al. (2005); Ontario; n = 390	104/390 = .267	116/390 = .297	.030	−.96	.34
(8) Stinchfield (2003); Minnesota; n = 1062	248/1059 = .234	256/1059 = .242	.008	−.41	.68

Endorsement rates for illegal acts criterion and the effect on diagnosis of GD based upon the absence or presence of the illegal acts criterion is shown in Table 6. The endorsement rate of illegal acts is zero or near zero for all eight community samples, and in the eight clinical samples the endorsement rate ranges from 19 to 67 %. When illegal acts criterion is removed, the number of individuals diagnosed with GD does not change in four of the eight datasets, and three datasets show one individual in each dataset no longer diagnosed with GD and one dataset has two individuals who lose their GD diagnosis. The removal of the illegal acts criterion changed the diagnosis of 5 out of 3247 individuals (.15 %).

**Table 6:** Endorsement rates for illegal acts criterion in clinical and community samples and effect of absence/presence of illegal acts criterion for diagnosis of GD

Source of data: Investigator, date, and sample size	Endorsement rate in clinical sample	Endorsement rate in community sample	Number diagnosed with GD with illegal acts criterion	Number diagnosed with GD without illegal acts criterion	Number of people no longer diagnosed with GD after illegal acts criterion was deleted
(1) Stinchfield et al. (2012); Ontario	66/133 = .50	0/222 = .00	215	215	0
(2) Stinchfield et al. (2012); Minnesota	52/92 = .57	1/120 = .01	113	113	0
(3) Jimenez-Murcia et al. (2012); Spain	43/232 = .19	0/50 = .00	204	203	1
(4) Jimenez-Murcia et al. (2009); Spain	53/284 = .19	1/283 = .004	277	277	0
(5) Stinchfield et al. (2007); Minnesota	53/87 = .61	2/43 = .05	90	89	1
(6) Stinchfield et al. (2007); Minnesota	76/150 = .51	0/25 = .00	147	146	1
(7) Stinchfield et al. (2005); Ontario	81/121 = .67	4/269 = .01	118	116	2
(8) Stinchfield (2003); Minnesota	135/259 = .52	1/800 = .001	256	256	0

## Discussion

The first research question was answered by computing reliability, validity and classification accuracy of the DSM-5 which found that the DSM-5 exhibited evidence of satisfactory reliability, validity and classification accuracy. The second question was answered by comparing DSM-5–DSM-IV on psychometric properties and the DSM-5 was similar to, and in some analyses better than, the DSM-IV. In terms of classification, the DSM-5 was slightly more accurate than DSM-IV, and the more significant error of false negatives, was reduced in the DSM-5, primarily due to the lowered threshold. The third question was answered by comparing the number of GD diagnoses without the illegal acts criterion to the number of GD diagnoses with the illegal acts criterion and it was found that five individuals, out of 3247, were no longer diagnosed with GD.

These results have three important diagnostic implications. First, lowering the threshold from five to four criteria had a modest improvement in diagnostic accuracy. Second, elimination of the illegal acts criterion had very little impact on diagnosis because most individuals who endorsed the illegal acts criterion had endorsed four or more of the other criteria and, therefore, had reached the DSM-5 threshold for diagnosis without the illegal acts criterion. Nevertheless, there were five individuals (out of 3247) who endorsed illegal acts but did not endorse four or more other criteria. Third, DSM-5 yields slightly higher GD prevalence rates than DSM-IV. This is largely due to the lower threshold from five to four criteria. Therefore, studies using DSM-IV diagnostic criteria are not directly comparable to studies using DSM-5 because of the lower cut score, the elimination of the illegal acts criterion, and the time frame of past 12 months in DSM-5.

In this study, the illegal acts criterion had the lowest endorsement rate of all ten diagnostic criteria in the clinical samples. Data from this study shows that a small number of individuals (5 out of 3247) lost their diagnosis of GD when the illegal acts criterion was eliminated. From a statistical standpoint, the elimination of illegal acts criterion has a very small impact on diagnostic accuracy because so few individuals are affected by it. From a clinical standpoint, it could be argued that this criterion should not be eliminated because it is known to be a symptom of GD. In recognition of these issues, the DSM-5 acknowledges illegal acts under the lying to others criterion where it states, “these instances of deceit may also include, but are not limited to, covering up illegal behaviors such as forgery, fraud, theft or embezzlement to obtain money with which to gamble (Criterion A7)” (APA 2013, p. 586). Thus, if clinicians and researchers inquire about illegal acts, and especially low threshold illegal acts such as “taking or borrowing” money from others to gamble without telling them, as examples of lying to others, then the effect of eliminating illegal acts as a stand-alone criterion will be attenuated.

These results indicate that, from a statistical standpoint, the illegal acts criterion can be eliminated with very little impact on diagnosis. There appears to be very few individuals who have engaged in illegal activity associated with their gambling and who have not already endorsed four or more other criteria.

While the DSM-5 showed modest improvements in diagnostic accuracy over the DSM-IV, the most important improvement is the lower false negative rate. From a clinical standpoint, false negatives are a more serious error than false positives. If someone who has GD is told that they do not have GD, this has the potential to result in serious consequences, such as continued gambling with adverse consequences. Lowering the threshold in DSM-5 reduced the false negative rate in all eight datasets, significantly in some datasets. In fact, reduction in the false negative rate could be justified even if the overall diagnostic accuracy was reduced, but in these datasets the false negative rate was reduced while maintaining high overall diagnostic accuracy that was as good as, or better than, the DSM-IV.

### Limitations

This study has some limitations. First, the clinical sample was made up of individuals in treatment and there are many individuals with a GD who are not represented in this sample. A significant number of problem gamblers who have committed crimes are in prison rather than in treatment (Turner et al. 2009, 2013). Therefore, these results need to be cross-validated in other samples and in different settings. Second, the reference standard used in these datasets of group membership in either gambling treatment or from the local community was devised to obtain a comparison between a group with and without PG. This method is imperfect and is only a proxy for a reference standard, given that there is no biological marker for the identification of a GD. The limitation is that there may be some individuals in the gambling treatment sample who do not have a GD and there may be some individuals in the community sample who do have a GD. Third, three of the DSM-5 criteria have minor revisions to the wording, such as inserting the word “often” in the preoccupation criterion, but the content and meaning of these criteria remain unchanged. These minor wording revisions could possibly influence whether a person endorses the criterion or not, but it is unlikely to substantively change the results of this study. These minor wording revisions in DSM-5 need to be included in diagnostic instruments from this point forward.

### Future Research Directions

Diagnostic criteria need to be further tested and cross-validated on larger and more diverse samples in a variety of settings (Gambino 2012). There is also the need to continue to generate additional diagnostic criteria. There may be other signs and symptoms that are better indicators of GD and therefore should be included in future revisions of the DSM. Also, some of the existing criteria are stronger indicators of GD than others and more research needs to be conducted on giving these items greater importance (weight) in the diagnostic process.

In summary, of the two revisions, the reduction of the cut score from five to four yields an improvement in diagnostic precision, particularly because it reduces the false negative rate, the more significant diagnostic error from a clinical standpoint. The elimination of the illegal acts criterion did not have much impact on diagnostic precision from a statistical standpoint due to the small number of persons who endorse this criterion and the even smaller number of persons who endorse this criterion who have not already endorsed four or more of the other criteria. By ensuring that illegal acts are addressed in the context of the lying to others criterion the effect of eliminating illegal acts as a stand-alone criterion can be minimized.

## Appendix: Stinchfield’s Measure of DSM-IV Diagnostic Criteria for Pathological Gambling

**Table:** Preface to all questions: in the past 12 months…

1. How often have you spent a lot of time thinking about past gambling experiences, planning your next gambling activity, or thinking of ways to get money to gamble?
2. How often have you needed to gamble with larger amounts of money or with larger bets in order to obtain the same feeling of excitement?
3. How often have you tried to control, cut back, or stop gambling several times and were unsuccessful? For example, setting a money or time limit for yourself and then going over it
4. How often have you felt restless or irritable when you tried to cut down or stop gambling?
5. How often did you feel that your gambling was a way of avoiding or escaping from personal problems or a way of relieving uncomfortable emotions, such as feelings of nervousness, helplessness, guilt, anxiousness or sadness?
6. After you lost money gambling how often did you return another day to get even or try to win back your losses?
7. How often have you lied to family members, therapists, or others to hide your gambling from them?
8. How often have you committed any illegal acts such as forgery, fraud, theft, or embezzlement to get money to gamble or to pay gambling debts?
9. How often have you risked or lost a relationship with someone important to you, or a job, or career opportunity because of gambling?
10. How often have you relied on others to pay your gambling debts or to pay your bills when you had financial problems caused by your gambling?
